# Supplementary material for: Contribution of Hydrogen Cyanide to the Antagonistic Activity of Pseudomonas Strains Against Phytophthora infestans
Source: Microorganisms. 2020 Jul 28;8(8):1144. doi: 10.3390/microorganisms8081144 (PMC7464445; doi:10.3390/microorganisms8081144)
Supplement: Supplementary file 1 [file microorganisms-08-01144-s001.pdf]

Supplementary information

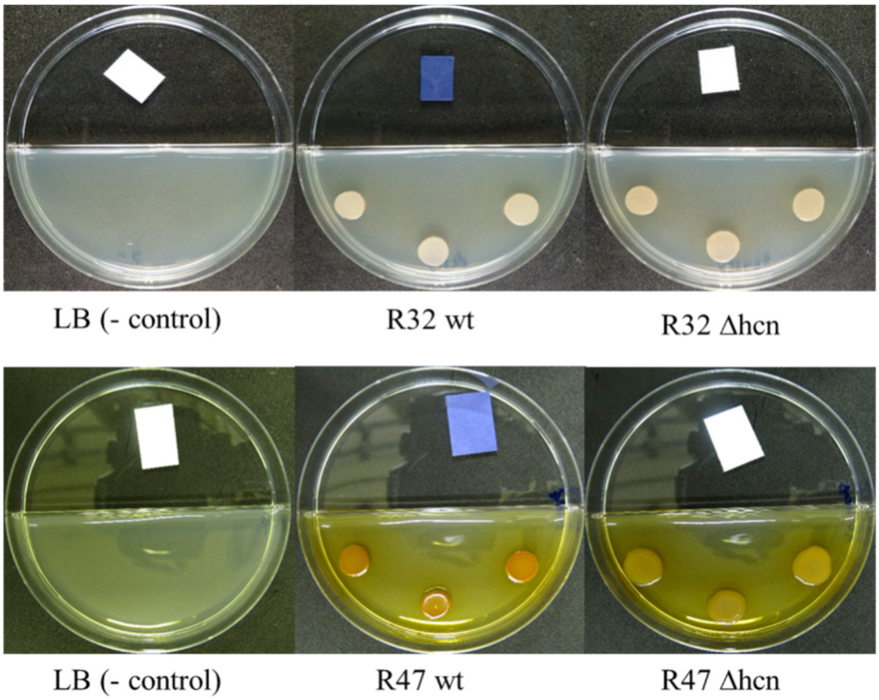

Figure S1. Cyanide detection assay. Bacterial strains were inoculated on v medium on one side, and detection paper was placed on the other empty side of split plate for 24 h at 30 °C. Images were taken after 24 h. Blue color represents the presence of cyanide in the surrounding. Uninoculated medium was used as control.

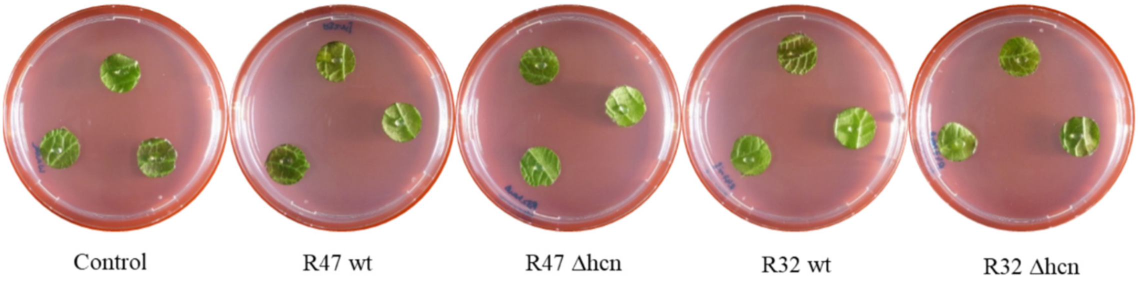

Figure S2. Representative images from potato leaf disc experiments where only *Pseudomonas* strains R47 (wt and Δhcn) and R32 (wt and Δhcn) were inoculated. Pictures were taken 6 days post-inoculation and incubation in humid chambers.

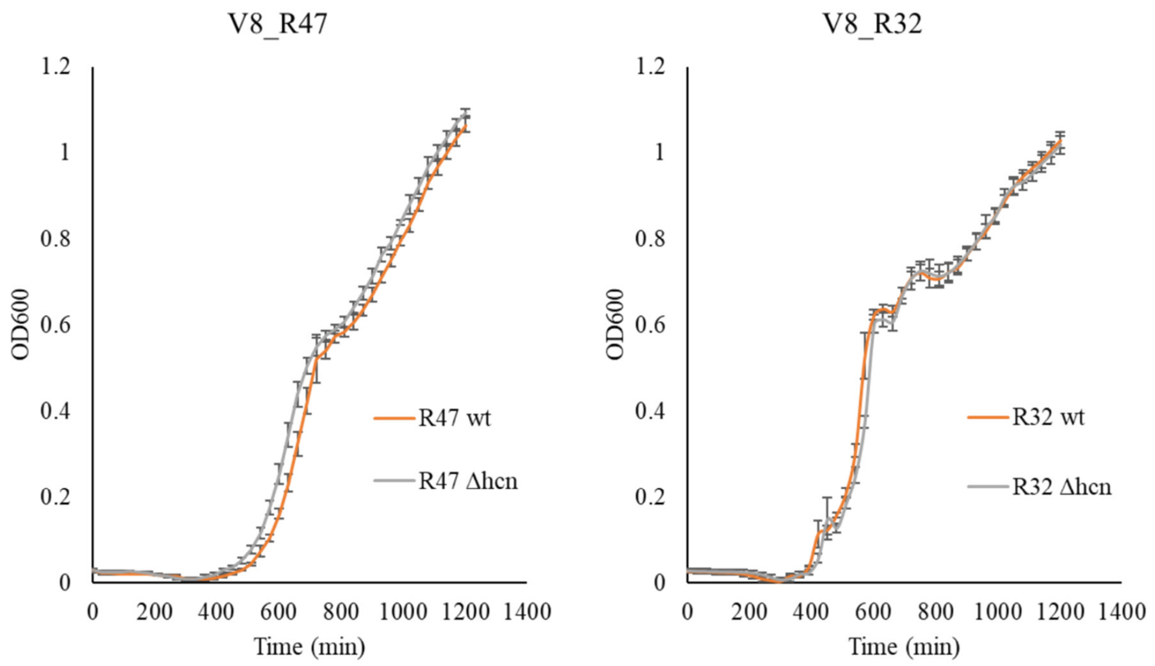

Figure S3. Growth curves for R47 (wt and Δhcn) and R32 (wt and Δhcn). Bacterial strains were grown in clarified V8 liquid medium for 20 h. Solid lines represent average of 3 replicates with corresponding error bars.

Table S1. Primers used in this study.

Name	Sequence; restriction sites (underlined)	Usage
hcn47-1	5′-CG<u>GAATTC</u>ACGTGCGCAGCCTGATCT-3′	Fragment 1 for R47
hcn47-2	5′-GG<u>GGTACC</u>GAGGGACTGGATGACGCT-3′	Fragment 1 for R47
hcn47-3	5′- GG<u>GGTACC</u>TTCCTCGCCGAGCGTTTC-3′	Fragment 2 for R47
hcn47-4	5′-CG<u>GGATCC</u>AGTCGGTGCAGATCTACTACG -3′	Fragment 2 for R47
hcn32-1	5′-CG<u>GAATTC</u>GCTGGCGTTGGTTGACAG-3′	Fragment 1 for R32
hcn32-2	5′-GG<u>GGTACC</u>GGGCTGGATATCTAGCTTTCG-3′	Fragment 1 for R32
hcn32-3	5′-GG<u>GGTACC</u>CTGGCCGAGCGTTTTGACTTG -3′	Fragment 2 for R32
hcn32-4	5′-CG<u>GGATCC</u>AAGCGTTGGTGGTTGCCCTGC-3′	Fragment 2 for R32

Table S2. Bacterial strains and plasmids used in this study.

Strain or plasmids	Relevant description	Reference
pEMG	Kanamycin^R, *ori* R6K, *lacZ* α, I-SceI sites	[1]
pSW-2	Gentamycin^R, *ori* RK2, *I-sce* I	[1]
pEMG::Δhcn_R47	pEMG plasmid containing 1 kb fragment 1-fragment 2 EcoRI-BamHI insert from R47 for deleting hcnABC	This work
pEMG::Δhcn_R32	pEMG plasmid containing 1 kb fragment 1-fragment 2 EcoRI-BamHI insert from R32 for deleting hcnABC	This work
R47 wild-type	wilt-type	[2]
R32 wild-type	wild-type	[2]
R47 pEMG::Δhcn	wild-type strain with pEMG::delhcnR47 plasmid, kmR	This work
R32 pEMG::Δhcn	wild-type strain with pEMG::delhcnR32 plasmid, kmR	This work
R47 Δhcn	Δhcn in-frame deletion mutant of R47 wild-type	This work

R32 Δhcn	Δhcn in-frame deletion mutant of R32 wild-type	This work

References

1. Martínez-García, E.; de Lorenzo, V. Engineering multiple genomic deletions in Gram-negative bacteria: Analysis of the multi-resistant antibiotic profile of Pseudomonas putida KT2440. *Environ. Microbiol.* **2011**, doi:10.1111/j.1462-2920.2011.02538.x.

2. Hunziker, L.; Bönisch, D.; Groenhagen, U.; Bailly, A.; Schulz, S.; Weisskopf, L. Pseudomonas strains naturally associated with potato plants produce volatiles with high potential for inhibition of Phytophthora infestans. *Appl. Environ. Microbiol.* **2015**, *81*, 821–830, doi:10.1128/AEM.02999-14.
